# QPRT: a potential marker for follicular thyroid carcinoma including minimal invasive variant; a gene expression, RNA and immunohistochemical study

**DOI:** 10.1186/1471-2407-9-93

**Published:** 2009-03-26

**Authors:** Nora Hinsch, Matthias Frank, Claudia Döring, Christian Vorländer, Martin-Leo Hansmann

**Affiliations:** 1 Senckenbergisches Institut für Pathologie, Klinikum der Johann Wolfgang Goethe-Universität Frankfurt am Main, Frankfurt, Germany; 2 Institut für Informatik, Johann Wolfgang Goethe-Universität Frankfurt am Main, Frankfurt, Germany; 3 Abteilung für Chirurgie, Bürgerhospital Frankfurt am Main, Frankfurt, Germany

## Abstract

**Background:**

The differential diagnosis between follicular thyroid adenoma and minimal invasive follicular thyroid carcinoma is often difficult for several reasons. One major aspect is the lack of typical cytological criteria in well differentiated specimens. New marker molecules, shown by poly- or monoclonal antibodies proved helpful.

**Methods:**

We performed global gene expression analysis of 12 follicular thyroid tumours (4 follicular adenomas, 4 minimal invasive follicular carcinomas and 4 widely invasive follicular carcinomas), followed by immunohistochemical staining of 149 cases. The specificity of the antibody was validated by western blot analysis

**Results:**

In gene expression analysis QPRT was detected as differently expressed between follicular thyroid adenoma and follicular thyroid carcinoma. QPRT protein could be detected by immunohistochemistry in 65% of follicular thyroid carcinomas including minimal invasive variant and only 22% of follicular adenomas.

**Conclusion:**

Consequently, QPRT is a potential new marker for the immunohistochemical screening of follicular thyroid nodules.

## Background

Differentiated thyroid carcinomas show an incidence of approximately 1% of all human malignancies [1]. In the group of endocrine malignant tumours they form, however, the largest entity. Differentiated thyroid carcinomas are a heterogeneous group composed of papillary, follicular (FTC) and medullary thyroid carcinoma [2]. In contrast to papillary carcinoma, which usually can be easily diagnosed by its characteristic growth pattern und nuclear features, FTC can appear cytologically identical to follicular thyroid adenoma (FTA). In these cases only the growth pattern distinguishes between benign and malignant thyroid tumours. According to the grade of invasion, FTC can be subdivided in widely invasive FTC and minimal invasive FTC. These show a different clinical behaviour [3]. Histologically, minimal invasive FTC as well as widely invasive FTC are usually well differentiated tumours, lacking cytological atypia. The diagnosis of FTC is based on histological findings such as angioinvasion and/or invasion that penetrates the full thickness of the tumour-surrounding capsule [4].

To what extend these criteria are fulfilled in special cases may remain a matter of interpretation and provides a high inter- and even intraobserver variability [5,6]. In order to establish additional criteria for FTC molecular techniques such as sequencing and FISH [7,8] were applied. These had limited value in discriminating FTC from FTA. RAS point mutations were evident in FTC as well as FTA, and chromosomal rearrangements (PAX8/PPARγ-rearrangement) were seen in some FTC and FTA with a preference of FTC [9-11].

The aim of our study was the discovery of new helpful immunohistochemical markers for the detection and definition of FTC.

## Methods

### Material

Tissue of 4 FTA, 4 minimal invasive FTC and 4 widely invasive FTC was divided in two parts each. One part of the specimens was fixed in 4% buffered formalin and embedded in paraffin. The other part was snapfrozen in liquid nitrogen and stored at -80°C.

qRT-PCR: Fresh frozen material from 4 FTA and 4 FTC was used.

Tissue from 149 patients was available for immunohistochemistry for a retrospective study. 77 of these showed FTA and 72 FTC. Huerthle cell tumours were not included in this study. Western Blotting was performed by using fresh frozen tissue of 3 FTC and 3 FTA. The tissue of these 3 FTC was also taken for gene expression analysis.

Moreover, a prospective study of QPRT-expression with staining of 149 solitary thyroid nodules was undertalen. Of these 149 nodules, 75 were FTA, 51 nodular goiter, 9 oxyphilic FTA, 7 minimal invasive FTC and 7 others (Graves' disease, papillary thyroid carcinoma, diffuse goiter, or no nodule).

All specimen were originally submitted for diagnostic purposes and studied in accordance with national ethical principles and in compliance with the Helsinki declaration. Informed consent for the use of fresh frozen material in gene expression analysis was obtained from the patients. The study was approved by the ethics committee of the university hospital Frankfurt/Main.

### RNA-extraction

RNA-extraction from fresh frozen tissue was performed using the RNAeasy Kit (Quiagen GmbH, Hilden, Germany) following the manufacturer's instructions. RNA quantity was measured using GeneQuant II photometer (Amersham Pharmacia Biotech, San Francisco, USA).

### Gene expression analysis

Four biological replicates of FTC and FTA were used for gene expression profiling. Briefly, DIG-labeled cRNA was generated using 1 μg total RNA per sample for amplification and labeling conducted according to manufacturer's instructions (Applied Biosystems RT-IVT Labeling Kit V.2.0 protocol). 10 μg of the DIG-labeled cRNA were hybridized on Applied Biosystems Human Genome Survey Microarrays V.2.0 according to manufacturer's instructions (Applied Biosystems Chemiluminescence Detection Kit protocol Rev. D). Raw data from our microarray experiments have been deposited in Gene Expression Omnibus: http://www.ncbi.nlm.nih.gov/geo/query/acc.cgi?acc=GSE15045

### Immunohistochemistry

Deparaffinized 5 μm paraffin sections were used for all immunostainings. For dermatopontin immunostaining antigen retrieval was performed (30 minutes cooking in citrate buffer (pH 6.0) in a microwave oven). For QPRT immunostaining, no antigen retrieval was necessary. The slides were treated with normal goat serum (4 μg/mL; Santa Cruz Biotechnology, Heidelberg, Germany), incubated at 4°C overnight applying the primary antibody dermatopontin (dilution 1:100, polyclonal antibody, Cat.Nr. 10537-1-AP, ProteinTech Group, Inc., Chicago, USA) or QPRT (dilution 1:200, clone 5D11, Abnova Corporation, Taipei, Taiwan). The Envision system with alkaline phosphatase and Fast Red (DAKO, Hamburg, Germany) was used. Tonsils with follicular hyperplasia where used as positive controls. For negative controls, no antibody was added.

The following criteria were applied to evaluate the immunostained sections:In positive cases, at least 10% of tumour cells had to show a cytoplasmatic immunoreaction. Most positive cases displayed positivity for more than 50% of the tumour cells. The staining pattern was heterogen. The percentage of positive cells was estimated in relation to negative tumour cells.

### TaqMan^® ^Quantitative real-time PCR

QPRT expression was analyzed by a quantitative real-time RT-PCR (qRT-PCR) assay (Hs00204757_m1, Applied biosystems, Weiterstadt, Germany). Beta-2-microglobulin (B2M) was used as endogenous control (4326319E, Applied Biosystems) for relative quantification. qRT-PCR was performed using a 96-well tray on the AbiPrism 7900 HT (Applied biosystems). The total reaction volume of 20 μl contained 1 × TaqMan universal Mastermix, 1 μl primer-probe mix and 5 μl cDNA. The samples were tested twice as singleplex-PCR. Results were specified as RQ-value, and calculated with SDS 2.2.1 (Applied biosystems) (RQ = 2^-ΔΔC^t. ΔΔCt is calculated as difference in ΔCt-value between the sample and the reference sample)

### Western Blot analysis

For western blotting snap frozen tumour tissue was lysed in 62.5 Tris-HCl, 2% SDS, 1.5% β-mercaptoethanol, 9% glycerol, 10 μg/ml leupeptin, 10 μg/ml aprotinin and 0.00125% bromophenol blue, cooked 10 min, with intermitting stirring, and cooled on ice.

Cell-lysates were subjected to a 10–20% SDS-PAGE (Criterion™ Pre-Cast Gel, Bio-Rad Laboratories GmbH, München, Germany) and electrotransferred onto a nitrocellulose membrane (Bio-Rad Immuno-Blot™ PVDF, Bio-Rad). The membranes were incubated with the QPRT antibody (dilution 1:1000) at 4°C overnight, followed by incubation with a horseradish-peroxidase-conjugated anti-mouse secondary antibody (dilution 1:2000, Code P0260, DAKO). For signal detection the chemiluminescence detection system ECL plus (Amersham Biosciences, Little Chalfont, UK) was used. Actin (C11, Santa Cruz Biotechnology) was used as loading control.

### Statistics

Statistical analysis was performed with the statistical computing environment R [12]. Additional software packages (ab1700, rma, multtest) were taken from the Bioconductor project [13].

Probe level normalization was conducted using the quantile normalization method [14].

Probeset summarization was calculated using the robust median polish method [15] on the normalized data. For each probeset an additive robust additive model on the logarithmic scale (base 2) was fitted across the arrays, considering the different affinities of the probes via the probe effect. We used a global filter to reduce the dimension of the microarray data: We applied an intensity filter (the signal intensity of a probe set should be above 100 in at least 25 percent of the samples, if the group size is equal) and a variance filter (the interquartile range of log_2 _intensities should be at least 0.5).

p-values were calculated applying the two sample t-test (assuming equal variances in both groups) to identify genes that are differentially expressed between the two groups. We use the False Discovery Rate (FDR) [16] to account for multiple testing. Also Fold Changes (FC) between the two groups were calculated for each gene. Differentially expressed genes were determined with p-value, FDR and FC criteria.

Unsupervised hierarchical cluster analysis was performed with the agglomeration method „average“. Manhattan method is used for the distance measure. Probe sets with a standard deviation more than one were included in the clustering. The results of immunohistochemical staining were analyzed with a Pearson's Chi-Square test, using SPSS 8.0 (SPSS Inc., Chicago, USA).

Sensitivity is defined as positive stained carcinomas in relation to all carcinomas included in the study. Specificity is the fraction of carcinomas in all positively immunostained samples.

## Results

### Gene expression analysis

**Figure 1:**
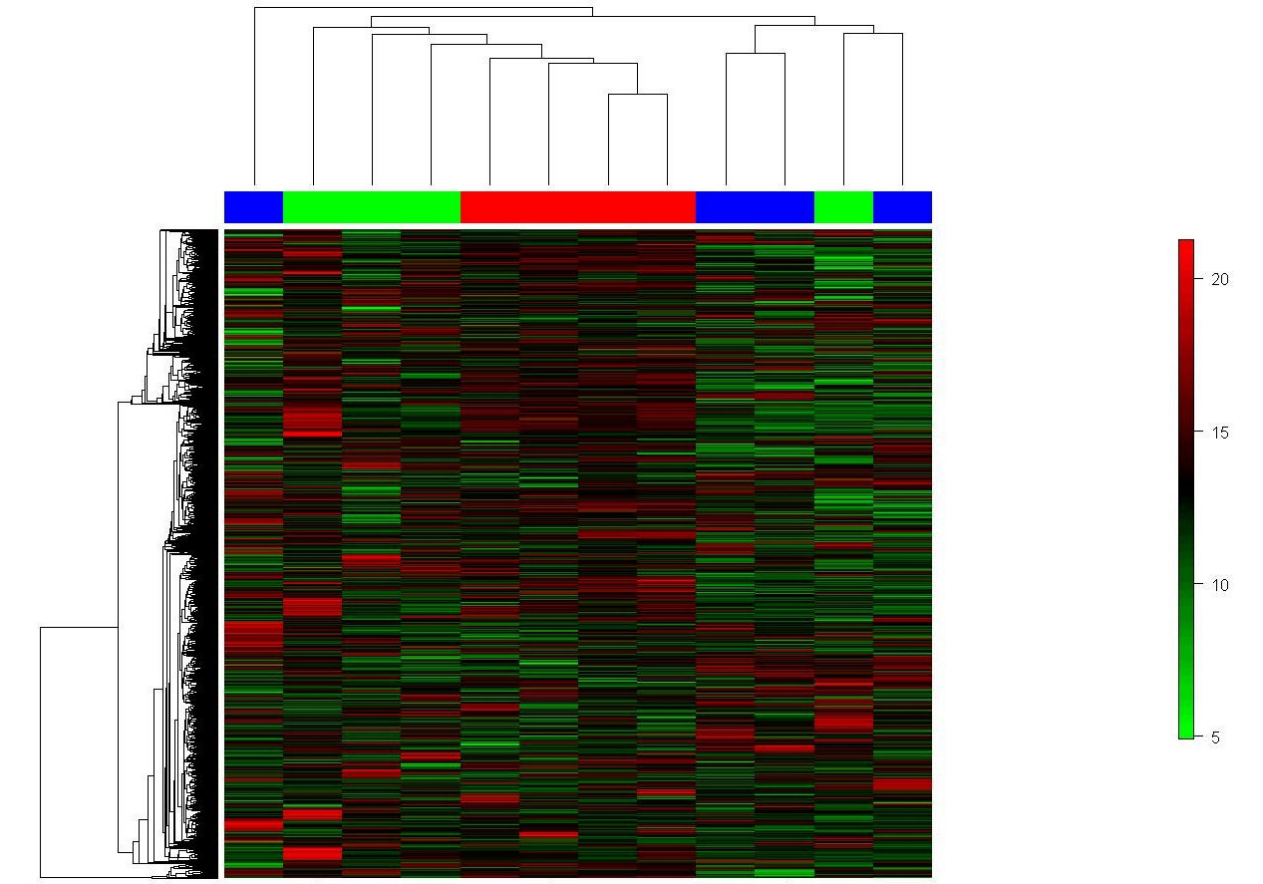
**Unsupervised clustering of 4 follicular adenomas (FTA), 4 minimal invasive follicular carcinomas (FTC) and 4 widely invasive FTC**. The clustering was performed with 3830 genes. Adenomas cluster to one group, which is surprisingly located between widely and minimal invasive follicular carcinomas. One of the widely as well as the minimal invasive follicular carcinoma is located in the each other group. Red: FTA, green: widely invasive FTC, blue: minimal invasive FTC

Gene expression analysis was performed using 4 FTA, 4 widely invasive FTC and 4 minimal invasive FTC. In unsupervised hierarchical clustering 3830 probe sets with a standard deviation more than one over all samples were included. The adenomas formed one group in the cluster analysis (figure 1) whereas widely invasive FTC and minimally invasive each FTC clustered together. An exchange between the two groups occurred once: one widely invasive FTC clustered with the minimal invasive FTC and one minimal invasive FTC clustered with the widely invasive FTC. Unexpectedly the widely invasive FTC clustered closer to the FTA than to the minimal invasive FTC. FTA in comparison to minimal invasive and widely invasive FTC differed in 25 genes (FC > 5 or <-5, p-value < 0.05, see Additional file 1). For further analysis the genes with the strongest differential expression, namely dermatopontin (FC 12.6 and 39.5) and quinolinate phosphoribosyltransferase (QPRT, FC -6.0 and -5.0) were selected.

### Immunohistochemistry

**Figure 2:**
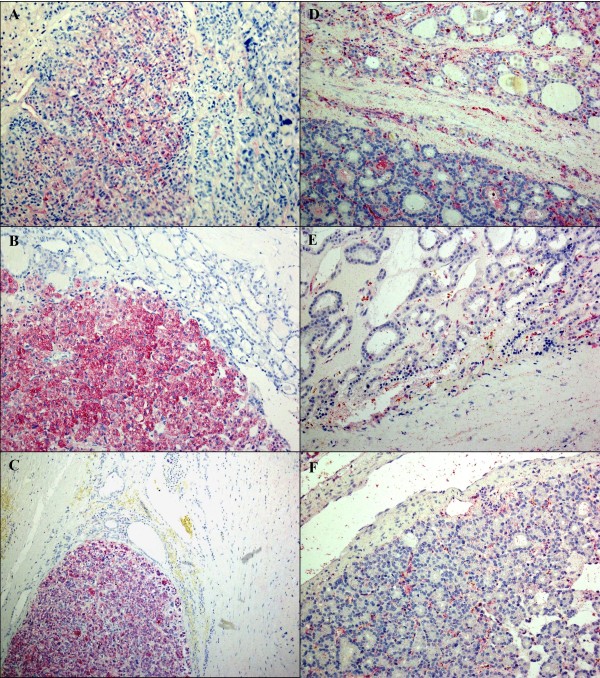
**Representative picture of immunohistochemical staining of FTC (A-C) and FTA (D-F)**. Carcinoma tissue displays a cytoplasmatic staining of the tumour cells, while adenoma tissue remains unstained.

Immunohistochemical validation of the results of the gene expression analysis was performed in a two-step-procedure: first, both antibodies were tested in a set of 32 probes. According to gene expression analysis, dermatopontin should have been positive in FTA and QPRT in FTC. However, dermatopontin appeared to be of little use (59% accuracy; 6 out of 17 positive FTA and 8 out of 15 positive FTC). Only QPRT was used with further 117 slides for a second step. Taken together, with QPRT a correct diagnosis according to our staining was reached in 107 out of 149 cases (72%). The sensitivity was 65% (47/72), the specificity 73% (47/64). The positive predictive value was 0,73, the negative predictive value was 0,71. Divided into subgroups, the sensitivity in minimal invasive FTC (60%) was lower than in widely invasive FTC (75%). Results are shown in table 1. Figure 2 shows an example of immunohistochemical staining.

**Table 1:** Tabular list of results of immunohistochemical QPRT staining.

	Staining		
	**+**	**-**	**Total**

**FTA**	17	60	77

**minimal invasive FTC**	29	19	48

**widely invasive FTC**	18	6	24

**Total**	64	85	149

Pearson Chi-Square = 28,4, p < 0,0001

In the prospective study, 59 out of 75 FTA were QPRT-negative (78,7%), while 6 out of 7 minimal invasive FTC were positive (85,7%). From the cases with nodular goiter, 14 out of 51 turned out to be positive (27,4%), most cases showed a focal positivity, often in areas with oxyphilic metaplasia. In accordance with this observation, oxyphilic FTA showed a positivity in most of the cases (6 out of 9, 66,7%). The other 7 cases included in this study were mostly negative (one oxyphilic FTC, two cases with Graves'disease, one papillary thyroid carcinoma, one diffuse goiter and one thyroid without nodules). Only one papillary thyroid carcinoma, follicullar variant stained positive.

### Western Blot

**Figure 3:**
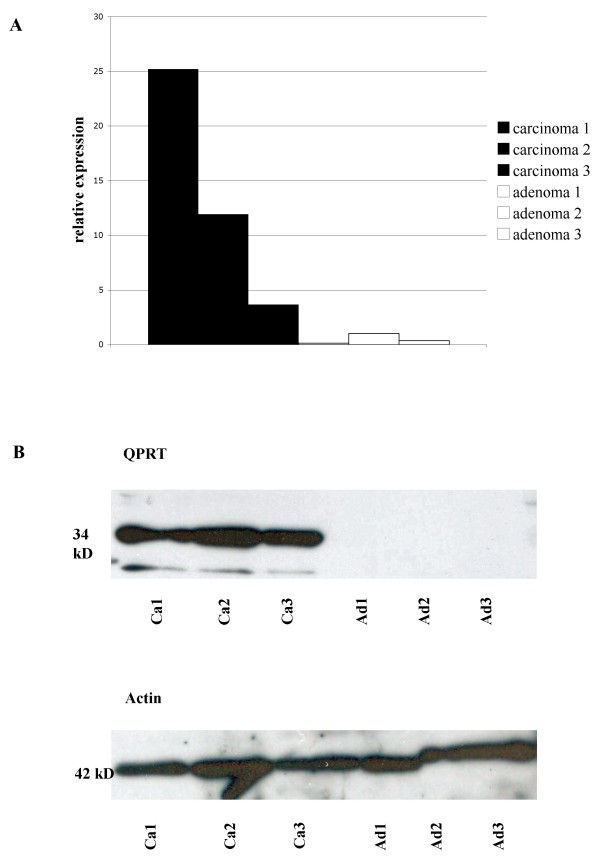
**qRT-PCR (A) and western blot analysis (B) of the 3 FTA and FTC presented in figure 2**. In qRT-PCR, carcinomas reveal a relative quantity of QPRT-RNA expression between 3,65 and 25,18. The relative quantity of QPRT-RNA expression in adenomas is between 0,13 and 1,00. In western blot analysis, FTC reveal a strong band at 34 kD, while follicular adenomas lack any band. Blotting with actin was performed as loading control. Ad: Adenoma; Ca: Carcinoma.

Western blotting confirmed the specificity of the QPRT-antibody. 3 FTA and 3 FTC were used for western blotting. Only the three FTC, but none of the FTA, showed a band at 34 kD (Figure 3B).

### qRT-PCR

qRT-PCR of FTC compared to FTA showed a ΔΔCt of 10,44 in FTC compared to 0,42 in FTA, indicating a nearly twenty times higher amount of QPRT-RNA in carcinoma tissue (Figure 3A).

## Discussion

We performed gene expression analysis of 12 follicular thyroid tumours (4 FTA, 4 minimal invasive FTC and 4 widely invasive FTC) resulting in a list of 25 genes which differed highly in their FTC and FTA expressions. The most interesting gene was QPRT showing a significant 5 fold upregulation in FTC compared to FTA. In further immunohistochemical validation QPRT could be established as helpful in discriminating between FTA and FTC. In a retrospective study 47 out of 72 FTC were positively immunostained by this antibody. 60 out of 77 FTA were negative for QPRT. Further validation was performed by using qRT-PCR and western blotting. Both validation methods confirmed our immunohistochemical findings. In an additional prospective study with 149 cases, 78,8% of FTA were stained negatively. 85,7% of minimal invasive FTC were stained positively with the QPRT-antibody. The potential value of this marker lies in the screening of thyroid nodules, with nodules staining positively being processed intensively.

QPRT is a 34 kD [17] key enzyme in the catabolism of quinolinate in the tryptophan-nicotinamide adenine dinucleotide pathway. Quinolinate is an endogenous neurotoxin [18], which is non-enzymatically derived from alpha-amino-beta-carboxymuconate-semialdehyde (ACMS) [19]. It has been speculated that QPRT activity increases in response to neurodegenerative events [20]. QPRT has been described in the nucleus and cytoplasma [21] of cells of the human central nervous system and the liver [22] and in blood cells (platelets and erythrocytes) [23]. Gene expression analysis revealed a QPRT upregulation with a fold change of 3,6 in uterine leiomyoma [24]. So far, QPRT has not been linked to other human diseases besides neurodegenerative disorders. In addition, it is unknown if QPRT is important in carcinogenesis. Until now QPRT has not been described in human thyroid tissue.

Different techniques such as global gene expression analysis and SAGE-analysis have been used to define follicular thyroid carcinoma (see Additional file 2). [25-30] The authors cited above validated the established gene expression or SAGE-results by qRT-PCR. qRT-PCR was also used as classifier to distinguish between a set of few genes [26,30] and gene trios [29], which were able to classify thyroid nodules correctly in between 83 and 96,7%. Two publications reported additional immunohistochemical staining based on some factors provided by gene expression analysis [26,30,31] resulting in 6 new immunohistochemical markers (see Additional file 2).

Of these markers, the two not commercially available antibodies integral membran proteine 1 (ITM1) and chromosome 1 open reading frame 24 (C1orf24) had the highest sensitivity of 100% [31]. Damage-inducible transcript 3 (DDIT3) and arginase II (ARG2) showed a high specificity with 85% positivity in carcinomas and only 9,4% positivity in adenomas [26].

Our gene expression results indicated, however, that no difference in gene expression between three of the four markers (ARG2, DDIT3 and ITM1), C1orf24 was present at our gene chip. One reason for this may be the different methodological approach. Cerutti et al. did SAGE-analysis of one FTC and one FTA. In contrast, we used the applied gene expression system at 12 follicular thyroid nodules. Furthermore, there was no statement of the number of minimal invasive FTC included in the study, and the total number of cases used for immunohistochemistry was limited to27 carcinomas and 22 adenomas.

Other factors obtained by gene expression analysis and tested immunohistochemically were cyclin D2 (CCND2) and protein convertase 2 (PCSK2). A combination of these two factors resulted in a sensitivity of 89,5% and a specificity of 80,8%.

Both factors were described as downregulated and consequently no immunostaining was found in FTC [30]. PCSK2 is, however, upregulated in FTC in gene expression analysis in two different studies [26,28]. Our global gene expression analysis also revealed a significant upregulation of PCSK2 in minimal invasive FTC compared to FTA. In addition, we found no significant difference in gene expression of PCSK2 between widely invasive FTC and FTA.

In several studies immunohistochemical markers for differentiating thyroid nodules were defined as independent from gene expression analysis. HBME-1, galectin-3 and CK19 were the markers most frequently referred to in literature and most commonly used for differentiating benign from malignant thyroid tumours. These markers showed, however, a low specificity for FTC. FTC was described as positive for HBME-1 in between 63 and 88%, while FTA was positive in 55,6 until 48,6% of the cases. Galectin-3 showed a positivity between 21 and 64% in FTC compared to 11 until 2,9% in FTA. CK19 also showed a low specificity with positivity in FTC in 21–44% and in FTA in 28,6 and 33% [32,23]. Therefore these markers seemed unpractical for routine diagnosis.

Our approach differs significantly from other studies in tissue selection. We grouped minimal invasive FTC from widely invasive FTC and FTA. Moreover we used only genes for further analysis, which were significantly differently regulated in the group of minimal invasive FTC and widely invasive FTC. Another major difference was the method used for gene expression. The articles mentioned above relied on SAGE-analysis, self-designed DNA-microarrays, with a number of genes between 3200 and 7458 [27-29], or affymetrix U95A or 133A oligonucleotid arrays [25,30]. We used oligonucleotide gene expression analysis from applied biosystems, which had, to our knowledge, not been used for analysis of follicular thyroid carcinoma before. This may explain why there was no overlap between the genes reported to be differently expressed as well as the set of genes we found. Another difference was the way of validating the gene expression analysis. We focussed on immunohistochemical staining, using a large set of follicular tumours. Therefore we concentrated on genes with high expression differences between FTC and FTA to find a gene suitable for immunohistochemical staining.

## Conclusion

In conclusion, gene expression analysis revealed a new immunohistochemical marker, which may be helpful in differentiating FTC from FTA. QPRT is a potential useful marker for immunohistochemical screening analysis of solitary thyroid nodules. In case of positivity the lesion should be processed extensively. This procedure probably reduces the number of misdiagnosed or overlooked minimal invasive carcinomas. The sensitivity of the new monoclonal antibody is, however, limited. For this reason, QPRT as other new markers has the highest diagnostic relevance if it is applied not solitary but in combination. Additional prospective studies of fine needle aspirates should be included.

## Competing interests

The authors declare that they have no competing interests.

## Authors' contributions

NH carried out the immunohistochemical, the real-time PCR studies as well as drafted the manuscript. MF carried out the gene expression analysis. CD performed the statistical analysis. CV gathered the material. MLH conceived of the study, and participated in its design and coordination and helped in drafting the manuscript. All authors read and approved the final manuscript.

## Pre-publication history

The pre-publication history for this paper can be accessed here:

http://www.biomedcentral.com/1471-2407/9/93/prepub

## Supplementary Material

Additional file 1 List of genes with a fold change >5 or <-5, and a p-value < 0,05. SDAd/SDCa_FC is the fold change of the gene expression in follicular thyroid adenoma (FTA) in relation to follicular thyroid carcinoma (FTC). SDAd/SDCami_FC is the fold change of the gene expression in FTA in relation to minimal invasive FTC. QPRT is the only gene in this list which is upregulated in FTC, the other genes are downregulated in FTC. Click here for file

Additional file 2 Excel file comparing different published studies concerning the discrimination of FTC and FTA using gene expression analysis. Click here for file
